# Editorial: Comorbidity and Autism Spectrum Disorder

**DOI:** 10.3389/fpsyt.2020.617395

**Published:** 2020-11-20

**Authors:** Manuel F. Casanova, Richard E. Frye, Christopher Gillberg, Emily L. Casanova

**Affiliations:** ^1^University of South Carolina School of Medicine Greenville, Greenville, SC, United States; ^2^Phoenix Children Hospital, Phoenix, AZ, United States; ^3^University of Gothenburg, Sahlgrenska Academy, Gothenburg, Sweden

**Keywords:** autism spectrum disorder, comorbidity, seizures, psychiatric disorders, gastrointestinal disorders, sleep disorders

This Research Topic consists of 32 articles, contributed by 283 authors, focusing on recent understanding regarding the impact and management of comorbidities associated with autism spectrum disorder (ASD). This Research Topic sought to answer questions such as: Are standard screening instruments capable of delineating the full range of impairment in ASD without considering comorbidities? Given the difficulties in communication for many ASD patients, what red flags point toward the presence of comorbidities? How do comorbid conditions relate to maladaptive behaviors? How do health care providers grapple with the juxtaposition of intellectual disability (ID), non-verbal clinical care, and comorbidities in ASD? This Research Topic attempted to answer these and other questions while raising awareness on how comorbid conditions increase both mortality and morbidity in ASD.

The presence of a chronic disorder that co-occurs, at the same time or in tandem, with a primary disease is a comorbidity. Defining the presence and natural history of comorbidities is important as it serves to better inform treatment and prognosis (1). For many individuals, comorbidities worsen the way they feel, behave, and think about themselves. Indeed, comorbidities add a multidimensional component to both diagnosis and treatment that colors the expectations of patients as well as their treating physicians.

By themselves, comorbidities are associated with worse outcomes and increased health needs. The presence of co-occurring medical conditions confers those affected with a higher level of morbidity, increased risk of depression, and reduced level of social well-being (2). These patients require more frequent and targeted clinical management (e.g., to avoid dangers of polypharmacy), services planning (e.g., coordination between various health care professionals), and financing. The more precise our understanding of a disease and its comorbidities the better the clinical care and health services that can be provided. It should come as no surprise that treatment of comorbidities generally improves the quality of life of affected patients.

The abundance of chronic conditions (e.g., diabetes, arthritis, obesity, depression) in modern medicine makes comorbidity the norm rather than the exception. In 2014, approximately 42% of adult Americans had multiple chronic conditions (3). Indeed, almost 77% of Medicare spending goes to people with multimorbid conditions; that is, the presence of 2 or more chronic disorders (4). A population-based survey in Sweden revealed that half of individuals with ASD (9-year-old twins born between 1992 and 2001) had four or more coexisting disorders while only 4% did not have a concomitant disorder (5). Considering that ASD is a lifelong condition, affecting 1 of every 54 children, the subject of comorbidities is of great importance for patient-centered health research, especially so as the mean age of our population increases (6–8).

In ASD, comorbidities do not occur evenly but tend to cluster into different subgroups. A recent study reported that ASD individuals can be subtyped according to whether they exhibit seizures, multisystem disorders, psychiatric disorders, or whether they lack a defined comorbidity (9). External validation of the different clusters was derived from within group commonalities in medical trajectories and an inability to generalize results among the different subgroups. The study demonstrates the usefulness of thinking about ASD as being divided into “essential or uncomplicated” and “complex” subgroups each with a different outcome and recurrence risk [e.g., (10)].

Comorbidities in ASD manifest as impairments outside of its core diagnostic features; the latter being deficits in social communication/interaction, and restricted, repetitive patterns of behavior, interests, or activities, including sensory processing difficulties (11). Epilepsy, psychiatric/behavioral complaints, and gastrointestinal (GI) disorders are common comorbidities of ASD, especially in subjects with intellectual disability (ID) (12). Comorbidities frequently manifest in preschool years (Muratori et al.) and their presence may be the best predictor of maladaptive behavior, respective of ASD symptom severity, presence of ID or limitations in adaptive functioning (Skwerer et al.).

The prevalence of comorbidities varies widely, from study to study, probably pointing to the fact that ASD is not a unitary disorder but a spectrum involving different pathophysiologies. Lumping patients into a single group tends to average out significant differences within a study population. Conclusions from these prevalence studies should be taken cautiously as research designs tend to exhibit multiple limitations. Indeed, when interpreting results, one must consider the variability ingrained in different ascertainment methods and the lack of available specialized consultative care in some study centers. In addition, many prevalence studies on comorbidities use small patient samples and/or fail to address the impact of demographic, socioeconomic, ethnic, and geographical factors on diagnosis.

In ASD, the expression of comorbidities stemming from any given organ system can take multiple forms. For GI comorbidities this may involve gastroesophageal reflux (GERD), constipation, diarrhea, food allergies, colitis, ulcers, and inflammatory bowel disease (13). These are all common disorders, readily observed in the general population and frequently associated with ID, sleep problems, behavioral disorders, and even connective tissue disorders like Ehlers-Danlos syndrome (EDS), which are also comorbid with ASD (Penzol et al.; Baeza-Velasco et al.). Associated behaviors may exhibit varying relationships to GI symptoms at different ages, a fact having important implications for medical care (Cawthrope; Ferguson et al.). In effect, treating GI disorders may help alleviate a broad array of disorders (e.g., mental, behavioral, sleep) in the multimorbid ASD patient (Neuhaus et al.).

Symptoms of comorbidities in ASD may be atypical and are often difficult to recognize (14). A major culprit propitiating these diagnostic difficulties is communication problems. In ASD, 25–50% of individuals are unable to speak (15). Furthermore, 90% of ASD toddlers are unable to point protodeclaratively or protoimperatively (16). Indeed, many individuals with ASD are incapable of pointing to the source of their discomfort, find it difficult to attend to or detect bodily sensations (17), have poor integration of body scheme representation, and have atypical sensory perceptions or reports of pain (18). This inability to communicate pain or discomfort to other people may propitiate the enactment of inappropriate behaviors as a way for patients to express themselves and attract attention to their plight.

Changes in behavior, even maladaptive behaviors, may indicate an underlying comorbidity. It is common for maladaptive behaviors to mask comorbidities and for these conducts to be dismissed as being either “autistic behaviors” (diagnostic overshadowing) or the result of environmental/sensory stressors. Changes in behaviors may manifest at home, at school, as alterations in every-day situations or as problems in already established personal relationships. Curiously, on some occasions, these behaviors may serve to mask an ASD diagnosis, especially in women. Females with ASD are the great chameleons of diagnostic medicine. They often gain social acceptance through rehearsed mimicry. Those that successfully camouflage their ASD are often misdiagnosed as having a borderline personality disorder, being bipolar or as suffering from major depression [see (19)]. In these cases, a putative comorbidity draws attention away from the primary diagnosis.

It is important to be cautious when facing unexplained multi-system involvement in a patient with ASD. Think of an underlying comorbidity if: there are unexplained signs and symptoms (including self-injurious and hetero-aggressive behaviors), in the case of maladaptive behaviors that cannot be contextualized during psycho-educational behavioral assessments (e.g., functional analysis), there are changes from baseline (including regression of skills—especially after 3 years of age), the patient is not responding as expected to therapeutic interventions, has a history of frequent visits to the emergency room, has a history of perinatal complications, or is taking multiple drugs along with over-the-counter (OTC) medications and dietary supplements (20–23). It is mandatory in these patients to screen for comorbidities by first taking a structured review of systems. It does not cost anything to ask questions! A review of systems is less expensive and more illuminating than many laboratory tests (24). It is surprising to us how many times a patient's symptoms are not reported because they are not considered relevant. In those occasions when parents do report symptoms, trust them, especially when they make comparisons to their other children.

Aging is a non-modifiable risk factor for comorbidity that makes apparent the need for comprehensive health care targeting multiple conditions. There are differences in the strength of association between symptoms across age strata that suggests a plausible temporal mechanism (Cawthrope). Gender is another non-modifiable factor that may help identify specific comorbidities (e.g., anorexia nervosa, see Margari et al.) or act through sex-related perinatal complications (25). Lack of exercise, improper diet (caloric dense and nutrient-poor), the use of certain medications (e.g., neuroleptics), food selectivity and genetic makeup may predispose individuals with ASD to obesity and poor nutrition (Nor et al.). This modifiable comorbidity, obesity, predisposes an individual to insulin resistance and type 2 diabetes mellitus, hypertension, dyslipidemia, cardiovascular disease, stroke, sleep apnea, gallbladder disease, hyperuricemia, gout, and osteoarthritis. Obesity has emerged as an epidemic among our ASD children who are plagued by both risk factors and an “obesogenic” environment (26). Prevention of excess weight gain is therefore a major target of personalized medicine meant to improve physical fitness in children with ASD (27–29).

This Research Topic can be divided into five subtopics that include: (1) comorbidities and psychiatric manifestations, (2) anatomical correlates, (3) specific examples, (4) intellectual disabilities, and (5) mechanisms. In the paragraphs below we will summarize each article within its subtopic.

1) Comorbidities and psychiatric manifestations: According to Druss and Walker (30) “having a mental disorder is a risk factor for developing a chronic condition, and having a chronic condition is a risk factor for developing a mental disorder.” Indeed, the pathways leading to the co-occurrence of medical and mental disorders are complex and bidirectional, often espousing common risk factors.

Using cross-sectional regression analyses (Avni et al.) found that an ASD diagnosis was associated with poorer adaptive functioning skills in a large and well-characterized sample of 260 young people with ADHD/ anxiety symptoms. The study also found that ADHD and anxiety symptoms predicted poorer adaptive functioning skills over and above IQ scores. Diagnosing these comorbidities early on is of importance in improving outcome.

Guerrera et al. recruited 37,500 children and adolescents and assessed psychopathological comorbidities using the Child Behavior Checklist (CBCL). Results showed that approximately 30% of the subjects exhibited internalizing problems as compared to the 6% that manifested externalizing problems. There was no correlation between CBCL scores and indices of ASD severity. The authors suggest that detection of distinct behavioral and emotional problems in ASD may lead to more specific and individualized treatments.

Muratori et al. completed a detailed assessment of the incidence of psychiatric comorbidity (PC) in 989 preschool age children with ASD using the DSM-Oriented Scales of an age-specific version of the CBCL 1.5–5. They compared IQ measures and other characteristics of the children with ASD only and ASD + PC, both mono-comorbid vs. multi-comorbid PC. They reported that 37.8% of the sample had at least one PC in addition to ASD, and that such subjects tended to exhibit lower overall performance IQ and higher ADOS calibrated severity scores. The higher rate of PC was reflective of increased Affective Problems, ADHD Problems, Anxiety Problems, and to a lesser extent Oppositional Problems. When considered together their data suggests that greater consideration of PC should be undertaken when evaluating and developing individual treatment or intervention plans for children with ASD.

Roberts et al. followed a large sample of males (*n* = 74) with fragile × syndrome (F × S) for their developmental and temporal profile of social avoidance during early childhood. Parameters for increased social avoidance predicted elevated ASD severity but reduced ADHD and anxiety within the study population. ASD, ADHD and anxiety symptoms related inconsistently to social avoidance behaviors. The results provide new insights into ongoing debates as to the independence or overlap among these disorders in F × S.

Skwerer et al. characterized the profile of co-morbid symptoms of psychopathology and emotion dysregulation in people with ASD with minimally verbal abilities. Correlations were found among ratings of emotional dysregulation, severity scores on co-morbidities and maladaptive behavior ratings, but none of these measures were correlated with ASD symptom severity scores. The number of clinically significant psychiatric symptoms was found to be the main predictor of maladaptive behaviors.

The study by Top et al. had three main aims. Firstly, the study reports data on self-report measures of sensory processing, anxious apprehension, and intolerance of uncertainty in three groups; (a) adults with ASD (AUT), (b) individuals with high levels of anxiety (ANX), and (c) neurotypical adults (NT). Adults with ASD scored higher than both other groups on Sensation Avoiding, Low Registration, and Sensory Seeking and scored similar to the ANX group on Sensory Sensitivity and Intolerance of Uncertainty. Anxious participants scored higher than both other groups on anxious apprehension. Significant correlations were reported between sensory sensitivity, anxious apprehension, and intolerance of uncertainty. Secondly, the study reports baseline physiological arousal in each of the three groups through measurements of baseline pupillary size. The AUT group had larger baseline pupil size than the NT group but similar size to the ANX group. However, the ANX group demonstrated decreasing pupil size throughout the experiment, which was not demonstrated by the AUT group. Thirdly and lastly, pupil size did not differ between groups in response to auditory stimuli or in habituation to stimuli over time.

Weiss and Fardella studied prior experiences with victimization and perpetration in adults with ASD (*n* = 45) and a healthy control group (*n* = 42). The participants filled out questionnaire measures, and the adults with ASD reported higher levels of negative experiences with victimization than the healthy control group. These group differences are not better explained by self-reported abilities in sociocommunicative abilities or emotion regulation difficulties. However, no group difference appeared in relation to experiences with perpetration. Previous studies have tended to focus on specific types of victimization and not perpetration.

2) Anatomical correlates: Form and function complement our understanding of each other. This is especially true for multimodal imaging classifiers that combine anatomical, neurochemical and white matter integrity measures in systems-biology-based models that enable health practitioners to better address the underlying causes of disease.

Cai et al. investigated structural differences between patients with High Functioning Autism (HFA) vs. Low Functioning Autism (LFA). Using an unbiased whole brain VBM-DARTEL method and a 3T MRI system to assess neuroanatomical differences in Gray Matter Volume (GMV) in various regions of interest (ROIs). They found that LFA and HFA had the same abnormal brain regions, but LFA had more abnormal brain anatomy regions than HFA. Increased GMV in the left Inferior Temporal Gyrus (ITG) were found in both HFA and LFA and increased GMV of the Left Medial Temporal Gyrus (MTG) was found only in the LFA group. Significant negative correlation between Gray Matter Volume of Left Inferior Temporal Gyrus and the score of repetitive behavior was also found, at least among HFA subjects.

Dekhil et al. reported on a computer-aided detection and diagnosis system (CAD) for ASD based on anatomical and functional indices of connectivity in brain regions that are commonly abnormal in ASD. The accuracy of a diagnosis was 75% when using fMRI data, 79% when using structural MRI data, and 81% when using a combination of both. By parcellating the data according to brain regions, a personalized brain map can be created that can help target therapeutic interventions.

Using functional magnetic resonance imaging (fMRI) (Lukito et al.) compared the neurofunctional correlates of duration discrimination between young adult males with ASD (*n* = 23), ADHD (*n* = 25), the comorbid condition of ASD+ADHD (*n* = 24), and those with typical development (TD, *n* = 26). By means of fMRI the authors aimed to study duration discrimination deficits and to test whether those deficits are underpinned by the same or different processes. Using both ROI and whole brain analyses the comorbid ASD + ADHD demonstrated significant under-activation in the right inferior frontal cortex compared to the other groups. The findings suggest that in adulthood, the comorbid ADHD + ASD, but not the pure conditions, demonstrate deficits in a brain region responsible for duration discrimination.

Nickel et al. reported a comparison of MRI findings in males with ASD and comorbid ADHD to those with ADHD only and neurotypical controls. A significant (after correction) decrease in volume of the left inferior frontal gyrus (pars orbitalis region) was found to be driven by ADHD, which was categorical rather than correlative with severity. ADHD alone, ASD alone, and ASDxADHD were each tied to trends in mean curvature changes in multiple different brain regions. The study suggested that the left inferior frontal gyrus might play a role in modulating symptoms of inattention and/or impulsivity in ADHD. Other observations based on cortical thickness and mean curvature, require further attention in studies using a larger sample population.

3) Specific examples: Feinstein first defined a comorbidity as, “Any distinct additional entity that has existed or may occur during the clinical course of a patient who has the index disease under study” (31). This terminology is imprecise and provides little guidance to health care professionals who may not find it self-evident as to which is the designated index condition and which is the comorbid condition (32). In the primary care setting index these labels are often neither obvious nor helpful, “and may vary in relation to the research question, the disease that prompted a particular episode of care, or of the specialty of the attending physician” [(32), p. 358]. Comorbidities challenge the single-disease framework upon which most of our health care system and medical education is configured (33).

Baeza-Velasco et al. described the current knowledge on possible links between ASD and joint-hypermobility related disorders. Joint hypermobility occurs in isolation or associated with genetic syndromes especially in collagen related disorders. Connective tissue diseases are frequently a source of joint pain and multisystem involvement. Individuals with ASD have social communication defects that may conceal pain conditions. The authors suggest keeping in mind joint hypermobility and/or collagen related disorders in people with ASD as a source of pain that can, in turn, exacerbate behavioral disturbances.

Cawthorpe screened for associations between ASD and a wide range of ICD diagnoses in a pediatric population. Analyses showed differences in terms of strength of associations across age strata suggesting plausible temporal mechanisms. Levels of GI symptoms accounted for unique variance in psychiatric outcomes over and above other factors, linking increased GI problems with increased psychiatric symptoms in children with ASD.

De Crescenzo et al. performed a meta-analysis of the literature on ASD symptoms in individuals with schizophrenia. Both disorders share impairments in reciprocal social interaction and social withdrawal, as well as other cognitive symptoms. Furthermore, childhood onset schizophrenia has a high comorbidity with ASD. This study compared ASD scores on the Autism Spectrum Quotient and subscores from 13 studies involving patients with Schizophrenia. Other ASD assessments were also included such as SRS, ADOS-2, CARS, and ASSQ. The evidence from 1958 patients supported the finding of increased ASD symptoms in patients compared to control samples, but fewer symptoms compared to those with ASD diagnosis. This suggest some shared psychiatric vulnerability, which is not surprising given some common genetic underpinnings.

Dy and Tanchanco provide the first case report of co-occurring achondroplasia and ASD. Both conditions are common on their own and their coincidental co-occurrence is to be expected. Achondroplasia typically presents with musculoskeletal impairments; however, the presence of delays in other domains of development, particularly social communication, should raise the suspicion of ASD. Recognition is important as early intervention is critical toward addressing the unique needs of such a patient.

Ferguson, Dovgan, Severns et al. studied the relationship between nutritional intake and gastrointestinal symptomatology in children with ASD (*n* = 120). The study found no significant associations between GI symptoms, omega-3 fatty acids and/or other micro- and macronutrients in the diet. The findings suggest a disconnect between dietary changes and GI symptoms in ASD individuals.

Ferguson, Hamlin, Lantz et al. examined the relationship between GI disorders, problem behaviors and internalizing symptoms in a sample of 340 children and adolescents with ASD. Externalizing problem behaviors and internalizing symptoms associated with GI problems differ between young and older children with ASD. The results suggest a different relationship of GI symptoms at different ages, a difference which may have implications for the longitudinal follow-up of patients as well as for their treatment.

Nor et al. designed and conducted a cross sectional study to assess the prevalence of obesity and overweight among Malaysian ASD children and adolescents. The methods were predominantly psychometric in nature and completed by the children's parents. The reported prevalence in ASD was higher than in the general population matched for age and gender, with higher risk for the older age ones, high maternal BMI, older paternal age, low physical activity, food refusal, and food selectivity. Feeding difficulties, limited variety, and a lack of physical activity was related to higher weight gain.

Lindor et al. studied whether the link between greater ASD symptom severity and problem behaviors is moderated by sleep disturbance. The authors reported a positive relationship between ASD severity and problem behaviors for those ASD individuals with mild or no sleep disturbance. By way of contrast, there was no significant relationship between ASD symptom severity and problem behavior among those ASD individuals with moderate-to-severe sleep disturbance. Significant problem behaviors were apparent across all individuals with moderate-to-severe sleep disturbances regardless of the severity of ASD symptoms. The study highlights how profiling of ASD individuals based on their sleeping habits may help identify those prone to clinically significant problem behaviors.

While sex-differences in ASD has been associated with IQ and cognitive function, Margari et al. examined whether male and female high functioning autism (HFA) patients might develop specific comorbidities phenotypes in this retrospective study. The study evaluated 159 HFA patients (100 male and 59 female) for the presence/absence, type and gender distribution of psychopathological comorbidities (Attention Deficit Hyperactivity Disorder, Anxiety Disorders, Depressive Disorders, Bipolar Disorder, Obsessive-Compulsive Disorder, and Anorexia Nervosa), according to DSM-5 diagnostic criteria. The study found a bias, favoring females, in Anorexia Nervosa among HF ASD patients.

Neuhaus et al. studied the records of 2,800 patients with ASD and found that parent-reported GI concerns are present in approximately one third of patients, and that GI symptoms are an independent predictor of internalizing, externalizing, and self-injurious behaviors. They document an increased prevalence of comorbid psychiatric problems in those patients with GI symptoms.

In a retrospective study Penzol et al. reviewed the prevalence of GI disorders in ASD and described their clinical correlates. The study included all patients (*n* = 845) with documented information regarding GI disorders (GID) admitted to a general hospital during a span of 3 years. GIDs were present in 30.5% of the patients, the most common complaint being constipation. GIDs were significantly associated with ID, sleep disorders and treatment with psychopharmacological drugs.

4) Intellectual disabilities: There is a large degree of clinical heterogeneity in both ID and ASD. Their complex interactions and expression may negate the generalizability of observational findings to the general population. Still, this area should be prioritized in research. Their multi-morbidity burden is greater, occurs at a much earlier age, and they are at the receiving end of health service inequalities.

Casanova et al. investigated genotype-phenotype correlations in ID. The study selected from a database of patients with ID and comorbidities of ASD, epilepsy, and a group without comorbidities (neither ASD nor epilepsy). These groups were further characterized by secondary manifestations of complex vs. simple facial dysmorphia (CFD/SFD) and neurodegenerative-like features (NLF). Phenotypic analysis showed high frequency of CFD in ID with ASD and NLF in ID with epilepsy. Gene covariation analyses were conducted and revealed ID networks with functional enrichment in relation to CFD and NLF. These findings suggest that clinical features related to ID are predictive of underlying genotype.

Miot et al. studied 63 adults with ASD-ID with detailed clinical examinations and screening for comorbidities. Investigators found a large range of comorbidities with a burden similar to those of older geriatric patients. This “premature aging” in adults with ASD-ID positively correlated with age, decreased autonomy, and polypharmacy. The results highlight the need for personalized medical care in this patient population.

Paulais et al. aimed to investigate possible cross-cultural differences and, on an individual level, the degree of heterogeneity in the cognitive and socio-emotional levels and severity of ASD symptoms. Developmental profiles of children with ASD + ID across countries showed extremely high heterogeneity in comparison with those of developmentally matched TD children. Profiles of ASD + ID children across countries were also similar to one another. ASD + ID children tended to have their lowest scores in language and vocal imitation. Heterogeneity within profiles was unrelated to country, but a few differences were found between countries in correlations of heterogeneity index with developmental level or developmental quotient. Algerian children had significantly greater differences between cognitive and socioemotional heterogeneity indices. Heterogeneity of profile was not related to age but was negatively related to overall developmental level and positively to severity of ASD.

5) Mechanisms: Many comorbidities represent intertwined conditions linked by shared risk factors (e.g., disease associated genes, biological pathways) and complications. The health profile of affected individuals is compounded by the duration, severity, and presence of other superimposed health conditions.

Ashwood reports that soluble TNFR2 levels are significantly decreased in ASD compared to TD controls following PHA stimulation, suggesting, lower TNF-alpha antagonism. Likewise, following PHA stimulation, cell surface TNFR2 was increased compared to controls. Overall, this work suggests that the TNF-alpha pathways, particularly that downstream of TNFR2, may be dysregulated in ASD.

Ferguson, Dovgan, Takahashi et al. sampled electrodermal activity (EDA) in eight individuals with severe ASD at a residential facility. The investigators found an anticipatory rise in EDA prior to problem behaviors. EDA may help anticipate and manage problem behaviors while also monitoring the return to baseline.

Hirosawa et al. reported higher rates of interictal epileptiform discharges (IEDs) in individuals with ASD compared to TD children. In addition, while IEDs have been reported to negatively impact cognition in TD children, little is known regarding the impact of IEDs in children with ASD. The study included 163 TD children and 107 children with ASD for which 10 min of MEG recording was conducted as well as an assessment of cognition with the Kaufman Assessment Battery for Children (K-ABC). Linear regression was employed to examine the effect of IED frequency on cognitive function. In the TD group, there was a significant negative relationship between mental processing (MPS) and IED frequency, while in the ASD group, a significant positive relationship was found between MPS scores and IED frequency. The authors suggest that the higher frequency of IEDs in ASD implies a local excitatory/inhibitory imbalance in the brain. However, the authors further suggest that this might not be pathogenetic in children with ASD, but instead might reflect epiphenomenal or compensatory processes.

Hughes and Ashwood report that antibodies that target *Candida albicans*, were positive in 36% of children with ASD and 14% of TD controls and GI dysfunction in half of the children with positive findings. The study draws attention to the fungal microbiota of individuals with ASD and the possibility of significant alterations (dysbiosis) in the same. The study provides suggestive evidence of a new microbial risk factor for ASD.

Sokol et al. review findings suggesting how secreted APPα and the ADAM family of α-secretases (non-amyloidogenic pathway of βAPP processing) may increase white matter volume in ASD. Recognition of those pathological pathway may lead to new treatment interventions.

Tye et al. considered those comorbid conditions that have been repeatedly associated with ASD; epilepsy, sleep, gastrointestinal, and immune functioning. The authors discuss research into potential etiological mechanisms and potential interactions between each comorbidity and ASD. The networks derived from the analysis of abnormal biological profiles may help define potential subgroups and personalize targets for interventions.

In conclusion, the presence of comorbidities delineates subgroups within ASD that allow us to better define underlying mechanisms, etiologies, and possible genetic/environmental contributions. Comorbidities provide additive risk factors for morbidity and mortality (Tye et al.) which demand special monitoring and long-term follow-up by primary care physicians. If health care providers are to improve outcome, it is imperative to reorient services in such a way so as to better recognize the presence of comorbidities and to refer patients to appropriate professionals. In order to diminish the burden of comorbidities in ASD, management requires a multidisciplinary team approach comprised by parents, special educators, psychologists, and medical specialists (34, 35). We also advocate for the implementation of a standardized review of systems, development of interpretative guidelines for polypharmacy peculiar to this patient population, execution of targeted assessments for specific psychiatric and GI disorders, and the enactment of preventative strategies for any existing modifiable risk factor (e.g., obesity).

## Author Contributions

All authors listed have made a substantial, direct and intellectual contribution to the work, and approved it for publication.

## Conflict of Interest

The authors declare that the research was conducted in the absence of any commercial or financial relationships that could be construed as a potential conflict of interest.
